# TUG1/MAZ/FTH1 Axis Attenuates the Antiglioma Effect of Dihydroartemisinin by Inhibiting Ferroptosis

**DOI:** 10.1155/2022/7843863

**Published:** 2022-09-17

**Authors:** Hailong Gong, Mingjun Gao, Yuancai Lin, Jinliang Liu, Zhiwen Hu, Jing Liu

**Affiliations:** ^1^Department of Neurosurgery, Shengjing Hospital of China Medical University, Shenyang 110004, China; ^2^Liaoning Clinical Medical Research Center in Nervous System Disease, Shenyang 110004, China; ^3^Key Laboratory of Neuro-Oncology in Liaoning Province, Shenyang 110004, China

## Abstract

Glioma is the most common primary intracranial malignant tumor in the brain. Currently, due to the limited treatment methods, the clinical outcome of patients with standard surgery combined with radiotherapy and chemotherapy is not satisfactory. Therefore, we urgently need to develop effective drugs to solve this problem. As a semisynthetic derivative of artemisinin, dihydroartemisinin (DHA) has been proved to have antitumor activity in glioma, which can induce apoptosis and inhibit the proliferation, migration, and invasion of glioma cells. In recent years, ferroptosis has been identified as another antitumor mechanism of DHA. Researchers have shown that DHA could promote ferroptosis in glioma cells. However, the specific molecular mechanisms of ferroptosis induced by DHA need more exploration. In this study, we found DHA could induce ferroptosis with ROS production and lipid peroxidation in glioma cells. Low expression of GPX4 and high expression of HMOX1 were identified in DHA treated glioma cells. Surprisingly, we found FTH1, a negative regulator of ferroptosis, upregulated in DHA treated glioma cells. It indicated that there should be some mechanisms that may cause ferroptosis attenuation in DHA treated glioma cells. For the first time, we confirmed that MYC-associated zinc finger protein (MAZ) could actively regulate FTH1 by binding to FTH1 promoter by CHIP assay. MAZ was further identified as the direct target of long noncoding RNA (lncRNA) TUG1 through luciferase assay. Downregulated expression of TUG1 and upregulated expression of MAZ were identified in DHA treated glioma cells. TUG1 overexpression or inhibition of FTH1 expression could enhance the antiglioma effect of DHA in vitro and in vivo, providing a promising strategy to enhance the antitumor effect of DHA in glioma.

## 1. Introduction

Glioblastoma (GBM) is the most common brain malignant tumor in adults, accounting for about 49.1%, and the median survival time is only 14.6-16.7 months. The current treatment standard for GBM includes surgical resection combined with radiotherapy and temozolomide chemotherapy, followed by maintenance of temozolomide for 6-12 months, but the treatment has little effect [1–3]. At present, tumor resistant mechanisms have been paid more and more attention which may attenuate the cytotoxicity of antitumor drugs. There is no doubt that searching methods to destroy the resistant mechanisms is a very promising strategy to enhance the antitumor effect.

Artemisinin, a sesquiterpene with a unique peroxide structure, is widely used in antimalarial treatment as the active component of *Artemisia annua* L [4]. Dihydroartemisinin (DHA) is the main derivative of artemisinin. In addition to its well-known antimalarial effects, DHA can also be used in the treatment of systemic lupus erythematosus, indicating its positive clinical application [5]. Furthermore, DHA has been shown to have antitumor activity in a number of human cancers, including colorectal, ovarian, liver, and pancreatic cancers [6–9]. Studies have shown that DHA can inhibit the migration, invasion, proliferation, and induce apoptosis of glioma cells [10–12]. In recent years, Chen et al. found that DHA could also induce ferroptosis in glioma cells [13]. However, the specific molecular mechanisms of ferroptosis induced by DHA still need more exploration.

Ferroptosis was discovered by Dixon et al. in 2012. It is characterized by changes in mitochondrial morphology, accumulation of lipid peroxidation products, and reactive oxygen species (ROS) produced by iron metabolism [14]. A growing number of studies have shown that ferroptosis is associated with many diseases, including neurodegeneration, tissue damage, inflammation, infection, and cancer [15–17]. Ferritin is the main iron storage protein in prokaryotes and eukaryotes, it is composed of 24 subunits of the heavy (FTH) and light (FTL) ferritin chains. A major function of ferritin is the storage of iron in a soluble and nontoxic state [18]. In recent years, some studies have shown that ferritin is a key regulator of ferroptosis, and its level changes affect the development of many diseases [19–21]. Low expression of FTH1 increases ferritin degradation and promotes ferroptosis [22]. However, we were surprised to find FTH1 expression was significantly increased in DHA treated glioma cells, which contradicts the fact that DHA promotes ferroptosis. Therefore, we considered there should be resistant mechanisms that may cause ferroptosis attenuation in DHA treated glioma cells. Besides, ferroptosis has been confirmed to play a part in therapies that reverse tumor resistance [23]. We wonder whether the antiglioma effect of DHA could be enhanced by increasing ferroptosis through attenuating the resistant mechanisms.

MAZ (MYC-associated zinc finger protein) is a zinc finger protein, which commonly expressed in different human tissues and regulates the expression of a variety of genes [24]. It regulates many pathways of cell proliferation, apoptosis, glycolytic, angiogenesis, migration, and invasion [25–27]. MAZ has also been extensively studied in gliomas. Current studies have shown that MAZ is related to the permeability of blood tumor barrier, angiogenesis, apoptosis, migration, and invasion of gliomas [24, 26–28]. Although whether MAZ is involved in ferroptosis is still unknown, we found there was a binding region between MAZ and FTH1 in the database. Based on the prediction, we further verified that MAZ may participate in ferroptosis by regulating the expression of FTH1.

Long noncoding RNAs (lncRNAs) participate in a wide range of biological functions through different molecular mechanisms. Some of them can bind to transcription sites and interact with proteins to regulate gene expression [29, 30]. We used the database and found lncRNAs that might bind to MAZ, and finally we focused on TUG1. TUG1 has been confirmed to be involved in angiogenesis, proliferation, apoptosis, migration, and invasion of glioma [31–34]. Although, until now there is no study showing TUG1 might be involved in ferroptosis, TUG1 has been identified to be associated with ROS [35]. Therefore, we wonder whether TUG1 might be involved in ferroptosis and whether it might participate in the antiglioma process of DHA through the regulation of MAZ.

For the first time, we demonstrated that upregulated FTH1 expression in DHA treated glioma cells which weakened the ferroptosis. Besides, we identified MAZ could target the promoter region of FTH1. Downregulated expression of TUG1 and upregulated expression of MAZ were confirmed in DHA treated glioma cells. Overexpression of TUG1 or inhibition of FTH1 expression enhances the antiglioma effect of DHA in vitro and in vivo, providing a promising method to enhance the antitumor effect of DHA in glioma.

## 2. Materials and Methods

### 2.1. Reagents and Antibodies

Dihydroartemisinin (HY-N0176) was obtained from MedChemExpress (Monmouth Junction, NJ, United States). DHA powder was dissolved to an initial concentration of 10 mM and stored at -80°C. Ferrostatin-1 (Fer-1, SML0583), Liproxstatin-1 (Lip-1, SML1414), and Deferoxamine (DFO, 252750) were supplied by Merck KGaA (Germany). CCK-8 Assay Kit (#CK04), ROS Assay Kit (#R252), and Cell Lipid Peroxidation Assay Kit (#L248) were all purchased from Dojindo (Japan). Penicillin and streptomycin were from Gibco (Thermo Fisher Scientific, United States). DMEM was purchased from CORNING (Corning Life Sciences, United States). Fetal bovine serum (TBS) was purchased from TBD Company (Tianjin, China). TRIzol and Lipofectamine 3,000 reagents were obtained from Life Technologies (United States). BCA Kit, SDS-PAGE Gel Kit, ECL Kit, and RIPA and PMSF buffers were obtained from Beyotime (Shanghai, China). The Dual-Luciferase Reporter Assay System was purchased from Promega (Beijing, China). The SimpleChIP Enzymatic Chromatin IP Kit was obtained from Cell Signaling Technology (CST, United States). The antibodies used were as follows: anti-GAPDH antibody (#10494-1-AP), anti-FTH1 antibody (#10727-1-AP), anti-SLC3A2 antibody (#15193-1-AP), anti-HMOX1 antibody (#10701-1-AP), anti-MAZ antibody (#21068-1-AP), and anti-GPX4 antibody (#67763-1-Ig) were obtained from Proteintech (Wuhan, China); antirabbit IgG (H+L), biotinylated antibody (#14708), and antimouse IgG (H+L), biotinylated antibody were obtained from Cell Signaling Technology (CST, United States). All antibodies are diluted according to the instructions.

### 2.2. Cell Lines and Transfection

U87 and U251 cell lines (human glioblastoma cells) were obtained from Shanghai Gene Chemistry (Shanghai, China). The Dulbecco's Modified Eagle Medium (DMEM) containing 10% serum and 1% penicillin-streptomycin, which was used for cell culture. The cells were cultured in an incubator containing 5% CO_2_ at 37°C. MAZ full length (MAZ(+)) plasmid and nontargeting sequence (negative control, NC) (MAZ(+)-NC); lncRNA TUG1 full length (TUG1(+)) plasmid and nontargeting sequence (negative control, NC) (TUG1(+)-NC) were synthesized (GenePharma, Shanghai, China). U87 and U251 were planted on 24-well plates, respectively, to establish stable transfected cell lines. Lipofectamine 3,000 reagent was used for plasmid transfection. When 80% confluence occurred, cells were transfected with the vectors: TUG1(+) or MAZ(+) plasmids. Suitable cells were screened by G418 and puromycin, and transfection efficiency was detected by qRT-PCR and Western blot.

### 2.3. Cell Viability Assay

Cell Counting Kit-8 (CCK-8) was used to detect the cytotoxicity of DHA in glioma cells. The two cell lines were inoculated into 96-well plates and treated with different concentrations of DHA for 48 h, respectively. CCK-8 reagent was mixed with DMEM in a ratio of 1 : 9, and 100 *μ*l of mixture was added to each hole. After incubating at 37°C with 5% CO_2_ for 1 hour, the cell viability was determined by measuring the absorbance at 450 nm with a microplate reader.

### 2.4. Detection of ROS Level

According to the ROS Assay Kit, two cell lines were inoculated in 6-well plates, treated with different concentrations of DHA for 48 h, then cleaned with PBS twice, added with the prepared working solution, and cultured in a 37°C incubator with 5% CO_2_ for 30 min. Finally, the cells were digested with trypsin and suspended in PBS, then the fluorescence signals were detected by flow cytometry.

### 2.5. Detection of Lipid Peroxides

According to the instructions of the lipid peroxide detection kit, U87 and U251 cells were inoculated in a six-well plate and incubated overnight in a 37°C incubator with 5% CO_2_. The cells were treated with different concentrations of DHA for 48 h, then the culture medium was removed, and the cells were washed with serum-free MEM medium twice. Add 300 *μ*l Liperfluo solution diluted with serum-free MEM medium in 1 *μ*mol/l to 6-well plates and incubate for 30 min in 5% CO_2_ incubator at 37°C. The solution was removed, cleaned with PBS twice, and observed under confocal microscope.

### 2.6. Quantitative Real-Time PCR

TRIzol reagent was used for cell lysis and RNA extraction from cells. The mRNA of TUG1, MAZ, FTH1, SLC3A2, GPX4, HMOX1, and GAPDH were reversely transcribed into cDNA by HiScript III RT SuperMix for qPCR Kit (Vazyme, Nanjing, China), ChanQ Universal SYBR qPCR Master Mix Kit (Vazyme) was used for staining. The mRNA expression was detected by a 7,500 Fast RT-PCR System (Applied Biosystems, USA). All primers used were purchased from Sangon Biotech and were shown in Table 1.

### 2.7. Western Blot Assay

The lysis buffer is a mixture of RIPA and PMSF (1 : 100) for cell lysis. The lysed cells were crushed with an ultrasonic crusher and centrifuged for 60 min (4°C, 5000 g). The concentration of supernatant was determined by BCA Kit. Protein samples were added to SDS-PAGE gel, and electrophoresis was performed at a voltage of 120 V. The samples were transferred to PVDF membrane and incubated with primary and secondary antibodies, respectively. ECL Kit was used to enhance the visualization of Western blot.

### 2.8. Dual-Luciferase Reporter Assays

The putative TUG1 binding site of the MAZ sequence (MAZ 3'UTR-Wt 5'-GCAGCCAGUGUCCCCCUCCCCUCUU -3') and mutation binding site of MAZ (MAZ 3'UTR-Mut 5'-UGUUGGUGGUAGGGGCGGGGGAGGAA-3') were amplified by PCR and cloned into a pmirGLO Dual-Luciferase Target Expression Vector (Promega, USA) to construct a luciferase reporter vector (GenePharma). HEK-293 T cells were inoculated on 96-well plates, and the cells were cotransfected with MAZ-Wt (or MAZ-Mut) and TUG1 (+) or TUG1(+)-NC plasmids using Lipofectamine 3000. After 48 h, luciferase activity was assessed using a Dual-Luciferase Reporter System.

### 2.9. ChIP Assay

The SimpleChIP Enzymatic Chromatin IP Kit was used for CHIP Assay. In simple terms, the cells were crosslinked with 1% final concentration of formaldehyde for 10 min at 37°C; Glycine was added into the petri dish and incubated at 37°C for 5 min to terminate cross-linking; The cells were cleaned twice with precooled PBS, scraped off, and collected in a centrifuge tube. According to the instructions, cells were lysed with lysis buffer and chromatin digested with micrococcal nuclease. The immunoprecipitate was incubated with anti-MAZ and normal rabbit IgG, respectively, and then gently shaken overnight with Protein G agarose beads at 4°C. Chromatin cleaning and elution follow instructions, 5 M NaCl and protease K were used to reverse DNA cross-linking, and CHIP DNA was purified finally. The obtained DNA was amplified by PCR using specific primers. Primer sequences are as follows: the experiment group of putative binding site MAZ in FTH1 promoter using the primers 5'-CAGTTCTTCCACCGATGC-3' and 5'-GTTAATGGCGGGTGACAG-3', generating a 193 bp product; the control group of putative binding site MAZ in FTH1 promoter using the primers 5'-GACAGCCTCAGAAGAACAT-3 and 5'-GTATGGTACTGGCACAGG-3', generating a 301 bp product.

### 2.10. Molecular Docking

The small molecular structure of DHA was obtained from TCSMP database (https://old.tcmsp-e.com/), and the crystal structure of FTH1, SLC3A2, HMOX1, and GPX4 was obtained from RCSB PDB database (https://www.rcsb.org/). The protein structure was further treated with PyMOL software (dehydrating and hydrogenation), the small molecular structure of DHA was docked with the protein structure of FTH1, SLC3A2, HMOX1, and GPX4 with Autodock software, then the docking site and binding energy were obtained. PyMOL software was used for visual processing and modification of the results.

### 2.11. Animal Experiment

All animal experiments were approved by the Animal Ethics Committee of Shengjing Hospital of China Medical University. All BALB/C nude mice were purchased from Huafukang Biotechnology (Beijing, China). These mice were 5 weeks old and weighed 14-16 g. We established subcutaneous tumour-forming mouse model by injecting 5 × 10^6^ U87 cells into the lateral abdomen of BALB/C nude mice. Animals were then treated with DHA solvent (50 mg/kg) by intragastric administration once a day for 26 days. The tumor volumes were measured every 2 days with calipers, and the calculation formula was (tumor length diameter × tumor short diameter^2^)/2.

### 2.12. Statistical Analysis

GraphPad Prism 8 was used to process statistical analysis and graphing of experimental results. All data were the results of at least three independent experiments. Our data were represented as mean ± SD. *P* < 0.05 was considered as statistically significant.

## 3. Results

### 3.1. The Antiglioma Effect of DHA Depends on Ferroptosis

**Figure 1:**
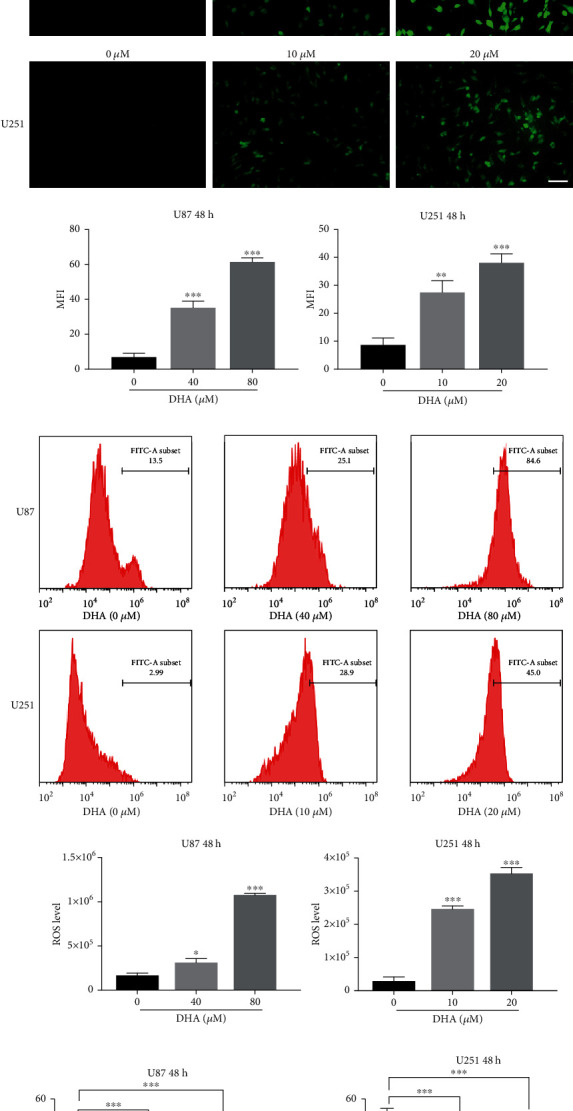
The antiglioma effect of DHA depends on ferroptosis. (a) The IC50 of DHA in U87 and U251 cells for 48 h. (b) The cytotoxic effect of DHA in U87 and U251 cells. (c) U87 and U251 cells were treated with different concentrations of DHA for 48 h, and the lipid peroxides (LPO) were detected by fluorescence microscopy. Scale bars: 20 *μ*m. (d) U87 and U251 cells were treated with different concentrations of DHA for 48 h, and the reactive oxygen species (ROS) were detected by flow cytometer. (e) The cell mortality rates were detected in DHA treated U87 (80 *μ*M) and U251 (20 *μ*M) cells for 48 h with or without Fer-1 (2 *μ*M) and Lip-1 (2 *μ*M). (f) U87 (80 *μ*M) and U251 (20 *μ*M) cells were treated with DHA for 48 h with or without DFO (100 *μ*M), and cell mortality rates were detected. ^∗^*P* < 0.05, ^∗∗^*P* < 0.01, and ^∗∗∗^*P* < 0.001; Data were mean ± SD from three independent experiments; *n* = 3 for all bar graphs.

Firstly, glioma cells U87 and U251 were treated with different concentrations of DHA (5, 10, 20, 40, 100, 200, 400, 600 *μ*M) for 48 h. The IC50 values of DHA at 48 h in U87 and U251 cells were 138.90 *μ*M and 26.76 *μ*M, respectively, (Figure 1(a)). According to the IC50 values of U87 and U251, we designed four different concentrations for each of them, to study. The results showed that the killing effect of DHA on U87 and U251 was dose-dependent (Figure 1(b)). The higher the concentration of DHA, the more obvious the tumor killing effect will be. We further examined some indicators of ferroptosis. Lipid peroxides (LPO) and reactive oxygen species (ROS) are commonly used indicators to detect ferroptosis. As shown in Figures 1(c) and 1(d), both LPO and ROS in U87 and U251 were significantly increased after DHA treatment. In addition, inhibitors of ferroptosis such as Deferoxamine (DFO), Ferrostatin-1 (Fer-1) and Liproxstatin-1 (Lip-1) were used to evaluate whether they affected the anticancer effects of DHA. The results showed that both Fer-1 and Lip-1 could reverse ferroptosis induced by DHA in U87 and U251 cells and attenuated the antiglioma effect of DHA (Figure 1(e)). Similarly, DFO can also significantly eliminate ferroptosis of DHA induced glioma cells (Figure 1(f)). These evidences indicated that DHA could exert its antiglioma effect depends on ferroptosis.

### 3.2. DHA Induces the Upregulated Expression of FTH1 That Attenuating Ferroptosis of DHA

**Figure 2:**
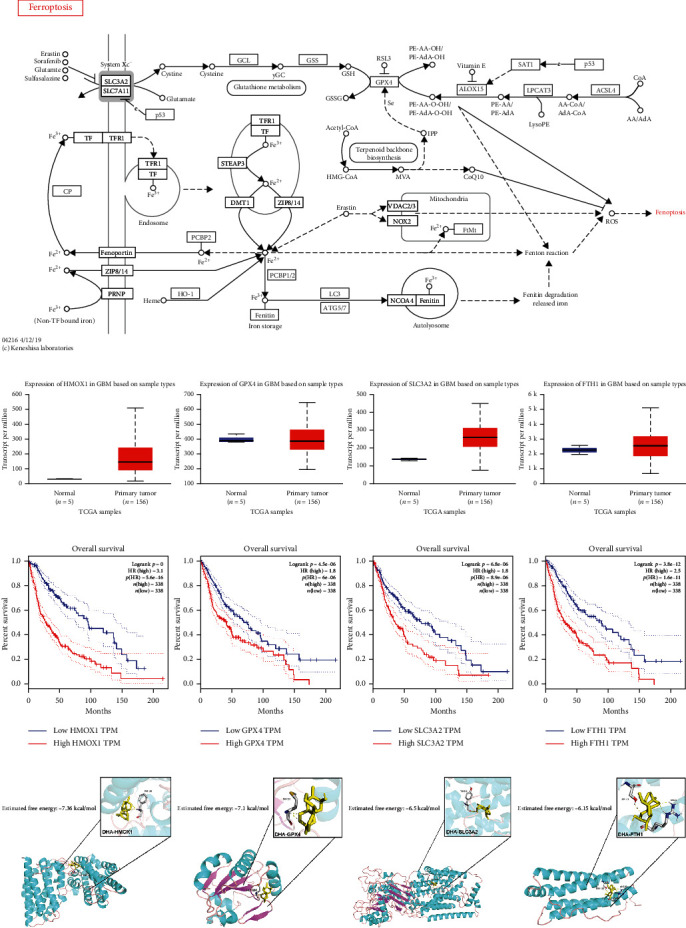
DHA induces ferroptosis by affecting the expression of some ferroptosis-related genes. (a) Ferroptosis map in KEGG PATHWAY Database. (b) The expression level of HMOX1, GPX4, SLC3A2, and FTH1 in GBM. (c) The prognosis analysis of HMOX1, GPX4, SLC3A2, and FTH1 in LGG and GBM. (d) Using AutoDock 4.2, the energy minimization structures of HMOX1, GPX4, SLC3A2, and FTH1 with DHA were analyzed by molecular docking.

**Figure 3:**
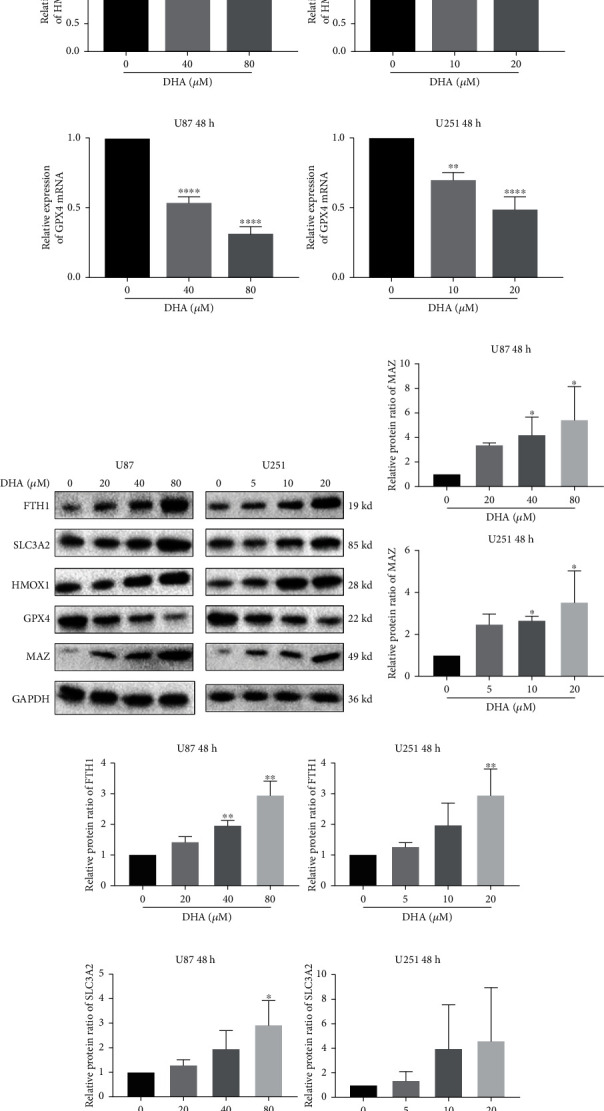
DHA induces the upregulated expression of FTH1 and SLC3A2. (a–d) The transcriptional expression levels of FTH1, SLC3A2, HMOX1, and GPX4 were detected in DHA treated glioma cells with different concentrations for 48 h by RT-qPCR. (e–i) Glioma cells were treated by DHA with different concentrations for 48 h. Total protein was extracted to disclose FTH1, SLC3A2, HMOX1, and GPX4 protein levels by western blot. ^∗^*P* < 0.05, ^∗∗^*P* < 0.01, ^∗∗∗^*P* < 0.001, and ^∗∗∗∗^*P* < 0.0001; Data were mean ± SD from three independent experiments; *n* = 3 for all bar graphs.

After confirming DHA can induce ferroptosis in glioma cells, we selected some related genes in the ferroptosis map of KEGG PATHWAY Database (Figure 2(a)). The expression levels of these genes in GBM and prognosis analysis in LGG (Figures 2(b) and 2(c)) were preliminarily obtained by using TCGA Database from UALCAN and GEPIA online website [36]. FTH1, SLC3A2, HMOX1, and GPX4 were firstly selected. We obtained the protein 3D structure of FTH1 (6B8F), SLC3A2 (6S8V), HMOX1 (1N45), and GPX4 (6HN3) from the Protein Data Bank website. AutoDock 4.2 software was used to conduct molecular docking between DHA small molecules (obtained from TCMSP database and analysis platform [37]) and the energy minimization structures of the above four genes. The results showed that the binding energies of DHA with FTH1, SLC3A2, HMOX1, and GPX4 were -6.15, -6.5, -7.32, and -7.1 kcal/mol (Figure 2(d)), respectively, suggesting that DHA may regulate the expression of them. Interestingly, we found that DHA significantly promoted FTH1, SLC3A2, and HMOX1 at transcription level (Figures 3(a)–3(c)) and protein level (Figures 3(e), 3(g)–3(j)), while significantly inhibited GPX4 (Figures 3(d) and 3(e)). Current studies have shown that HMOX1 was a positive regulator, while FTH1, SLC3A2, and GPX4 were negative regulators of ferroptosis [20, 38–40]. These results suggested that the low expression of GPX4 and high expression of HMOX1 induced by DHA might promote ferroptosis in glioma cells, while the high expression of FTH1 and SLC3A2 might reverse this process. In the following study, we selected FTH1 as our key research target.

### 3.3. MAZ Promotes FTH1 Expression by Targeting the Promoter Region of FTH1

**Figure 4:**
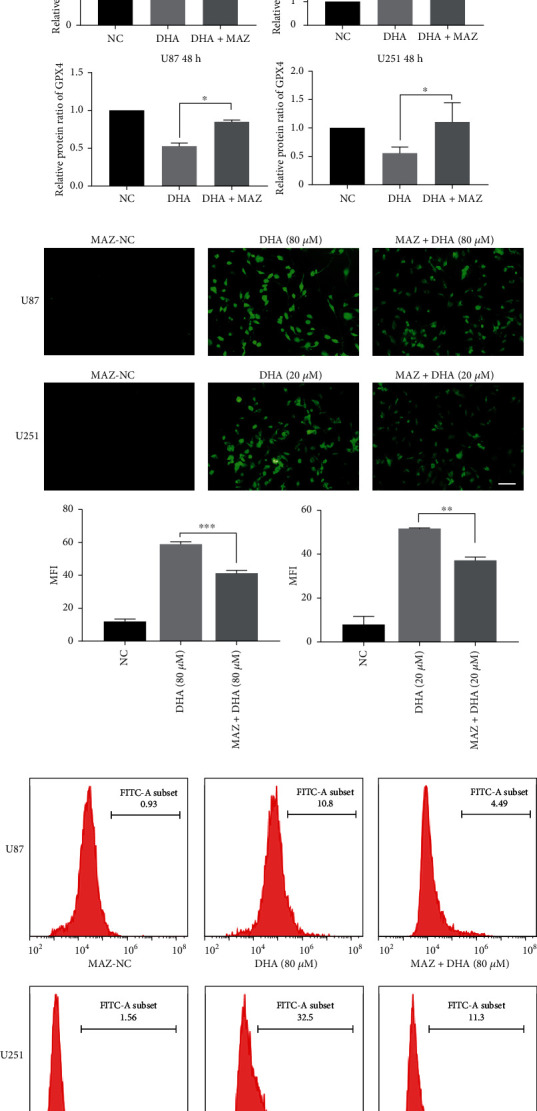
MAZ promotes FTH1 expression by targeting the promoter region of FTH1. (a) The transcriptional expression level of MAZ in U87 and U251 cells were detected after DHA treatment with different concentrations for 48 h by RT-qPCR. (b) MAZ targeted the promoters of FTH1 in U87 and U251 cells. Transcription start sites (TSS) and putative MAZ binding sites are illustrated. Immunoprecipitated DNA was amplified by PCR. Normal rabbit IgG was used as a negative control. (c) RT-qPCR and Western blot were used to detect the overexpression efficiency of MAZ at transcription level and protein level. (d) The protein expression levels of FTH1 and GPX4 in glioma cells were detected after DHA treatment with or without MAZ overexpression by Western blot. (e) LPO in glioma cells were detected after DHA treatment with or without MAZ overexpression by fluorescence microscopy. (f) ROS in glioma cells were detected after DHA treatment with or without MAZ overexpression by flow cytometer. (g) Cell mortality rates in U87 and U251 cells were detected after DHA combined with DFO treatment with or without MAZ overexpression. (h) Cell mortality rates in U87 and U251 cells were detected after different concentrations of DHA treatment with or without MAZ overexpression. ^∗^*P* < 0.05, ^∗∗^*P* < 0.01, ^∗∗∗^*P* < 0.001, and ^∗∗∗∗^*P* < 0.0001; Data were mean ± SD from three independent experiments; *n* = 3 for all bar graphs.

We used Human TFDB Database (Human Transcription Factor Database) and hTFtarget Database (Human Transcription Factor Targets Database) to screen and predict the upstream transcription factors of FTH1, and ZNF384 and MAZ were selected as our primary targets. Thus we verified their expression levels in DHA treated glioma cells by qRT-PCR and Western blot. The results showed that ZNF384 had no significant changes in transcription level (Supplementary Figure 1), while MAZ (Figures 3(f) and 4(a)) was significantly increased in transcription level and protein level. Further, we predicted potential binding sites between MAZ and FTH1 promoter region through the Bioinformatics database (JASPAR). Consistently, CHIP Assay showed that MAZ was directly associated with the putative binding site of FTH1 (Figure 4(b)). Next, we constructed stably MAZ overexpressed transfection strains for U87 and U251 cell lines, and evaluated the transfection efficiency by qRT-PCR and Western blot (Figure 4(c)). We found the expression of FTH1 was more obviously increased in DHA treated MAZ overexpression group than DHA treated alone group. Meanwhile, the downregulated expression of GPX4 in DHA treated alone group significantly reversed in DHA treated MAZ overexpression group (Figure 4(d)). The levels of LPO and ROS in DHA treated MAZ overexpression group were lower than those treated by DHA alone (Figures 4(e) and 4(f)). Besides, MAZ overexpression increased the inhibitory effect of DFO on ferroptosis in DHA treated glioma cells (Figure 4(g)). Moreover, we found the effect of MAZ overexpression weakened the cytotoxicity of DHA in U87 and U251 (Figure 4(h)). These results indicate that MAZ overexpression attenuates ferroptosis induced by DHA in glioma cells by promoting the expression of FTH1.

### 3.4. TUG1 Targets MAZ and Regulates Ferroptosis Induced by DHA

**Figure 5:**
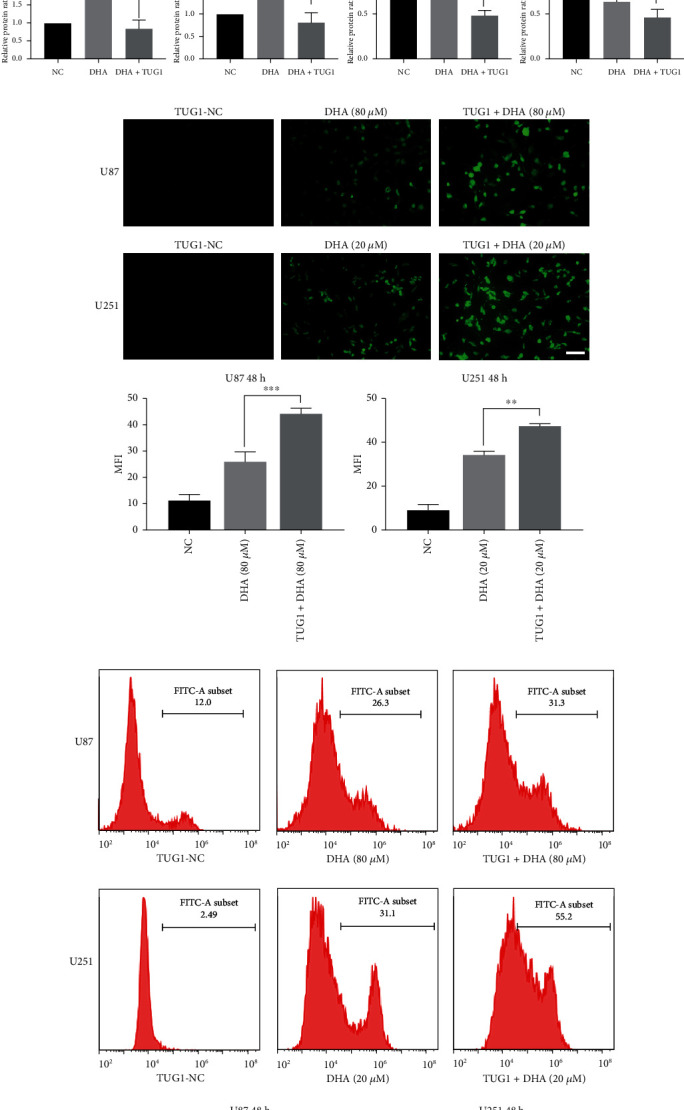
TUG1 targets MAZ and regulates ferroptosis induced by DHA. (a) The expression level of TUG1 in U87 and U251 cells were detected after DHA treatment with different concentrations for 48 h by RT-qPCR. (b) Dual-luciferase reporter assay was performed to determine the binding sites of TUG1 and MAZ 3′-UTR. (c) Western blots were performed to determine the protein expression levels of FTH1, GPX4, and MAZ in glioma cells after DHA treatment with or without TUG1 overexpression. (d) LPO was detected after DHA treatment with or without TUG1 overexpression by fluorescence microscopy. (e) ROS in glioma cells was detected after DHA treatment with or without TUG1 overexpression by flow cytometer. (f) Cell-mortality rates in U87 and U251 cells were detected after DHA combined with DFO treatment with or without TUG1 overexperssion. (g) Cell-mortality rates in U87 and U251 cells were detected after different concentrations of DHA treatment with or without TUG1 overexpression. ^∗^*P* < 0.05, ^∗∗^*P* < 0.01, ^∗∗∗^*P* < 0.001, and ^∗∗∗∗^*P* < 0.0001; Data were mean ± SD from three independent experiments; *n* = 3 for all bar graphs.

Combined with RNAInter Database (RNA Interactome Database) and literature, we selected TUG1, LINC00467, COX10-AS1, and FGD5-AS1 as our possible candidate genes which may regulate MAZ. Their expression levels in DHA-treated U87 and U251 cells were verified by qRT-PCR (Figure 5(a) and Supplementary Figure 2). The results showed that the expression levels of LINC00467 and FGD5-AS1 were not significantly changed, while the expression levels of TUG1 and COX10-AS1 were significantly downregulated. Then, we predicted the binding positions and binding energies of TUG1 and COX10-AS1 to MAZ by using IntaRNA in RNAInter database. Since TUG1 has been shown to be associated with ROS, we firstly selected TUG1 as our research object and predicted that TUG1 might bind to the 3 ‘UTR of MAZ mRNA through a specific sequence Alu element, with a binding energy of -47.1002 kcal/mol. Consistently, the Luciferase Reporter Assay confirmed this. As shown in Figure 5(b), the luciferase activity in the MAZ wild-type (WT)+TUG1 group was significantly lower than that in the MAZ wild-type (WT)+TUG1 negative control (NC) group, while the luciferase activity in the MAZ mutant (Mut) group was not affected. After construction of TUG1-overexpressed U87 and U251 glioma cells, as expected, the expression of MAZ and FTH1 at the protein level was significantly downregulated (Figure 5(c)). In DHA-treated glioma cells, LPO and ROS were significantly increased combined with TUG1 overexpression (Figures 5(d) and 5(e)). In addition, TUG1 overexpression also weakened the inhibitory effect of DFO on ferroptosis in DHA treated glioma cells (Figure 5(f)). These results showed that overexpressed TUG1 could enhance ferroptosis induced by DHA in glioma cells. Meanwhile, we further verified that TUG1 overexpression could enhance the cytotoxicity of DHA in U87 and U251 (Figure 5(g)).

### 3.5. TUG1/MAZ/FTH1 Axis Participates in the Antiglioma Effect of DHA by Regulating Ferroptosis

**Figure 6:**
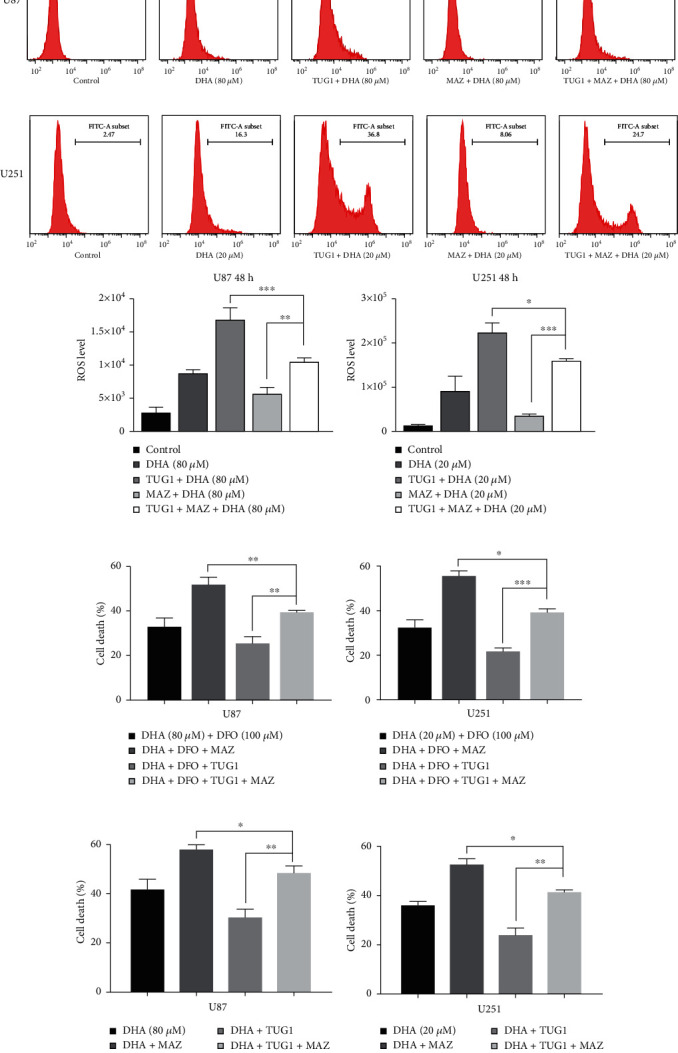
TUG1/MAZ/FTH1 axis participates in the antiglioma effect of DHA by regulating ferroptosis. (a–b) ROS in U87 and U251 cells were detected after DHA treatment with TUG1 overexpression alone, MAZ overexpression alone, and both TUG1 and MAZ overexpression. (c–d) Cell mortality rates in U87 and U251 cells were detected after DHA combined with DFO treatment with TUG1 overexperssion alone, MAZ overexpression alone, and both TUG1 and MAZ overexpression. (e–f) Cell mortality rates in U87 and U251 cells were detected after different concentrations of DHA treatment with TUG1 overexpression alone, MAZ overexpression alone, and both TUG1 and MAZ overexpression. ^∗^*P* < 0.05, ^∗∗^*P* < 0.01, and ^∗∗∗^*P* < 0.001; Data were mean ± SD from three independent experiments; *n* = 3 for all bar graphs.

As shown in Figures 6(a) and 6(b), after DHA treatment, ROS level in glioma cells with both TUG1 and MAZ overexpression was lower than TUG1 overexpression alone, but higher than MAZ overexpression alone. These results indicate that TUG1 overexpression can attenuate the inhibitory effect of MAZ overexpression on ferroptosis. Consistently, we add DFO to U87 and U251 treated with DHA. The results showed that TUG1 overexpression can increase the mortality of DHA treated glioma cells, which can be reversed by MAZ overexpression, confirming the resistant mechanism by this pathway (Figures 6(c) and 6(d)). Finally, we observed the effects of TUG1 overexpression alone, MAZ overexpression alone, and both TUG1 and MAZ overexpression on cytotoxicity of DHA in glioma cells (Figures 6(e) and 6(f)). These results showed TUG1 overexpression could significantly enhance the cytotoxicity of DHA in glioma cells, however the enhanced effect was attenuated by MAZ overexpression. It was concluded that TUG1/MAZ/FTH1 axis participates in the antiglioma effect of DHA by regulating ferroptosis.

### 3.6. TUG1 Overexpression Enhances the Antiglioma Effect of DHA In Vivo

**Figure 7:**
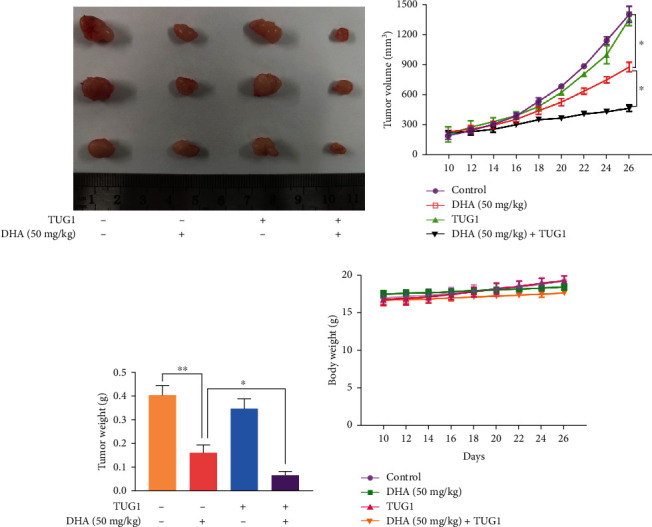
TUG1 overexpression enhances the antiglioma effect of DHA in vivo. (a) Subcutaneous transplanted tumor sizes of nude mice in each group. (b) Changes in the volumes of subcutaneous transplanted tumors in each group for 26 days. (c) Weights of subcutaneous transplanted tumors in each group were shown. (d) Mice body weights were measured. ^∗^*P* < 0.05 and ^∗∗^*P* < 0.01; Data were mean ± SD from three independent experiments.

The U87 cells were implanted into the lateral abdomen subcutaneous tissue of nude mice, and divided into groups of control, DHA, TUG1 overexpression, and DHA+TUG1 overexpression. The results showed the tumor volumes and weights in the DHA+TUG1 overexpression group were the smallest and lowest, suggesting TUG1 overexpression could significantly enhance the antiglioma effect of DHA (Figures 7(a)–7(c)). In addition, there was no significant difference in body weight between the NC group and each treatment group (Figure 7(d)).

## 4. Discussion and Conclusion

The prognosis of glioblastoma is the worst among the intracranial tumors, and until now there is still no effective method to increase the survival duration. Besides the effectiveness of drugs, we should also pay attention to the toxicity of drugs which may limit the use in clinical patients [41]. Thus, searching the potential antitumor drugs which have already been applied in clinical other diseases would be more promising [42, 43]. Natural products with anticancer activities have received increasing attention in the past few years due to their excellent safety and efficacy [11, 43, 44]. Dihydroartemisinin (DHA) is an antimalarial drug that has been widely used in clinic. In addition, its anticancer effects have attracted more and more attention. DHA plays an anticancer role through a variety of molecular mechanisms, such as inducing apoptosis, inhibiting proliferation, tumor metastasis, and angiogenesis [45]. Moreover, a new mechanism of DHA against glioma has gradually come into view, that was ferroptosis [13]. However, there are few reports about this, and more mechanisms need to be explored.

Ferroptosis is a regulated form of cell death, characterized by the iron-dependent accumulation of lipid peroxidation to a lethal level. The essence of ferroptosis is glutathione depletion, the activity of glutathione peroxidase (GPX4) decreases, and lipid peroxides cannot be metabolized through the glutathione reductase reaction catalyzed by GPX4. After that, Fe^2+^ oxidizes lipid to produce reactive oxygen species (ROS), which promotes ferroptosis [46]. System XC cysteine/glutamate reverse transporter, consisting of a light chain subunit (xCT, SLC7A11) and a heavy chain subunit (SLC3A2), is responsible for intracellular uptake of cysteine in exchange for intracellular glutamate. Inhibition of xCT system expression induces ferroptosis [47]. Heme oxygenase 1 (HMOX1) has also been identified as a gene associated with ferroptosis, and upregulation of HMOX1 can promote ferroptosis [40]. Ferritin heavy chain (FTH1) is a component of ferritin, and downregulation of FTH1 can mediate the degradation of ferritin and promote the occurrence of ferroptosis [22]. In order to further determine the specific pathway of DHA-induced ferroptosis in glioma cells, we used Western blot to detect the changes in protein levels of these ferroptosis-related genes. The results showed that GPX4 expression was significantly decreased, and HMOX1 expression was significantly upregulated after DHA treatment. These results suggest that DHA can promote ferroptosis in glioma cells by downregulating GPX4 and upregulating HMOX1. Interestingly, FTH1 and SLC3A2 were significantly upregulated, which was contrary to our expectations, suggesting that there should be some resistant mechanisms which made the upregulation of FTH1 and SLC3A2 attenuating ferroptosis induced by DHA in glioma cells. We selected FTH1 as our research object, and further identified which mechanism that upregulated the expression of FTH1 and weakened ferroptosis.

In this study, we used JASPAR database to predict the binding sites of FTH1 with possible transcription factors. And we found there was a binding site between MYC associated zinc finger protein (MAZ) and FTH1 (CACCCCTCCGG) with a correlation score of 0.92. CHIP Assay further verified that MAZ could target the promoter region of FTH1 through our predicted binding sites. MAZ is a widely expressed transcription factor and is closely related to the occurrence and development of various cancers [25]. Then we identified MAZ was significantly upregulated at transcription and protein levels in DHA treated glioma cells. Current studies have shown that MAZ involved in the proliferation, migration, invasion, glycolysis, and angiogenesis of cancer cells [25, 27, 48, 49]. However, it is not clear whether MAZ is involved in ferroptosis. Base of those above, we constructed U87 and U251 with MAZ overexpression, and observed the changes of ferroptosis induced by DHA through detecting LPO and ROS in glioma cells. The results showed the levels of LPO and ROS in DHA treated MAZ overexpression cells were lower than those treated by DHA alone. Meanwhile, MAZ overexpression increased the inhibitory effect of DFO on ferroptosis in glioma cells. These results suggest MAZ overexpression can attenuate ferroptosis induced by DHA in glioma cells. Besides, we found the effect of MAZ overexpression weakened the cytotoxicity of DHA in U87 and U251. These results indicate that MAZ/FTH1 axis takes part in the regulation of ferroptosis, which affects the antiglioma effect of DHA.

Combined with RNAInter Database, we tried to find the upstream regulation mechanisms of MAZ, then TUG1 caught our attention. We predicted that TUG1 might bind to the 3 ‘UTR of MAZ mRNA through a specific sequence Alu element. Further, we used Luciferase Reporter Assay to confirm this. Current studies have shown that TUG1 was involved in the proliferation, migration, invasion, apoptosis, and angiogenesis of glioma cells [31–33, 50]. Besides, Gong et al.‘s study showed a regulatory relationship between TUG1 and ROS [51]. Thus we wonder whether TUG1 may participate in ferroptosis by regulation of MAZ/FTH1 axis in DHA treated glioma cells. The results showed that TUG1 overexpression can attenuate the inhibitory effect of MAZ overexpression on ferroptosis. TUG1 overexpression could enhance ferroptosis and increase the mortality of DHA treated glioma cells.

**Figure 8:**
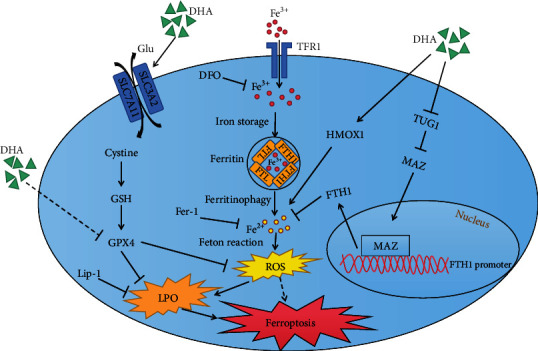
A proposed hypothesis shows the ferroptosis induced by DHA in glioma cells.

Here, we reported that there was a resistant mechanism in ferroptosis induced by DHA which attenuates its antiglioma effect (Figure 8). For the first time, we identified MAZ could target the promoter region of FTH1 and confirmed TUG1 could bind to the 3 ‘UTR of MAZ mRNA. Activating TUG1/MAZ/FTH1 axis obviously enhanced the antiglioma effect of DHA by increasing ferroptosis, providing a promising method to enhance the antitumor effect of DHA in glioma.

## Figures and Tables

**Table 1:** Primers sequence for Real-Time qPCR.

Gene	Forward primer	Reverse primer
TUG1	ACCTGGACCTGGAACCCGAAAG	GTGCTGGTGGTAGTGCTTGCTC
COX10AS1	CCGCTACACCAAGTCAGGAACATC	GTTGCTCACTCCTTGCCTCACTC
ZNF384	GTCTCAGGTCAGATCGAGAACA	ACTCTGTGTCCATACTGATGCC
MAZ	GCCCTTCAAATGTGAGAAATGT	ACCTTCATGTGGTCCGAAATAT
FTH1	CCCCCATTTGTGTGACTTCAT	GCCCGAGGCTTAGCTTTCATT
SLC3A2	TGAATGAGTTAGAGCCCGAGA	GTCTTCCGCCACCTTGATCTT
GPX4	GAGGCAAGACCGAAGTAAACTAC	CCGAACTGGTTACACGGGAA
HMOX1	AAGACTGCGTTCCTGCTCAAC	AAAGCCCTACAGCAACTGTCG
GAPDH	GGAGCGAGATCCCTCCAAAAT	GGCTGTTGTCATACTTCTCATGG

## Data Availability

The database and online site presented in the study are included in the article/supplementary materials, and further inquiries can be directed to the corresponding author.

## Supplementary Materials

Supplementary Materials The supplementary figures referred to this article are included in the Supplementary materials. The transcriptional expression levels of ZNF384 and COX10-AS1 after DHA treatment are shown in Supplementary Figures 1 and 2, respectively.
