# Supplementary material for: TUG1/MAZ/FTH1 Axis Attenuates the Antiglioma Effect of Dihydroartemisinin by Inhibiting Ferroptosis
Source: Oxid Med Cell Longev. 2022 Sep 17;2022:7843863. doi: 10.1155/2022/7843863 (PMC9509247; doi:10.1155/2022/7843863)
Supplement: Supplementary Materials — The supplementary figures referred to this article are included in the Supplementary materials. The transcriptional expression levels of ZNF384 and COX10-AS1 after DHA treatment are shown in Supplementary Figures 1 and 2, respectively. [file 7843863.f1.docx]

**Supplementary Materials**

**Supplementary Figure 1**

**
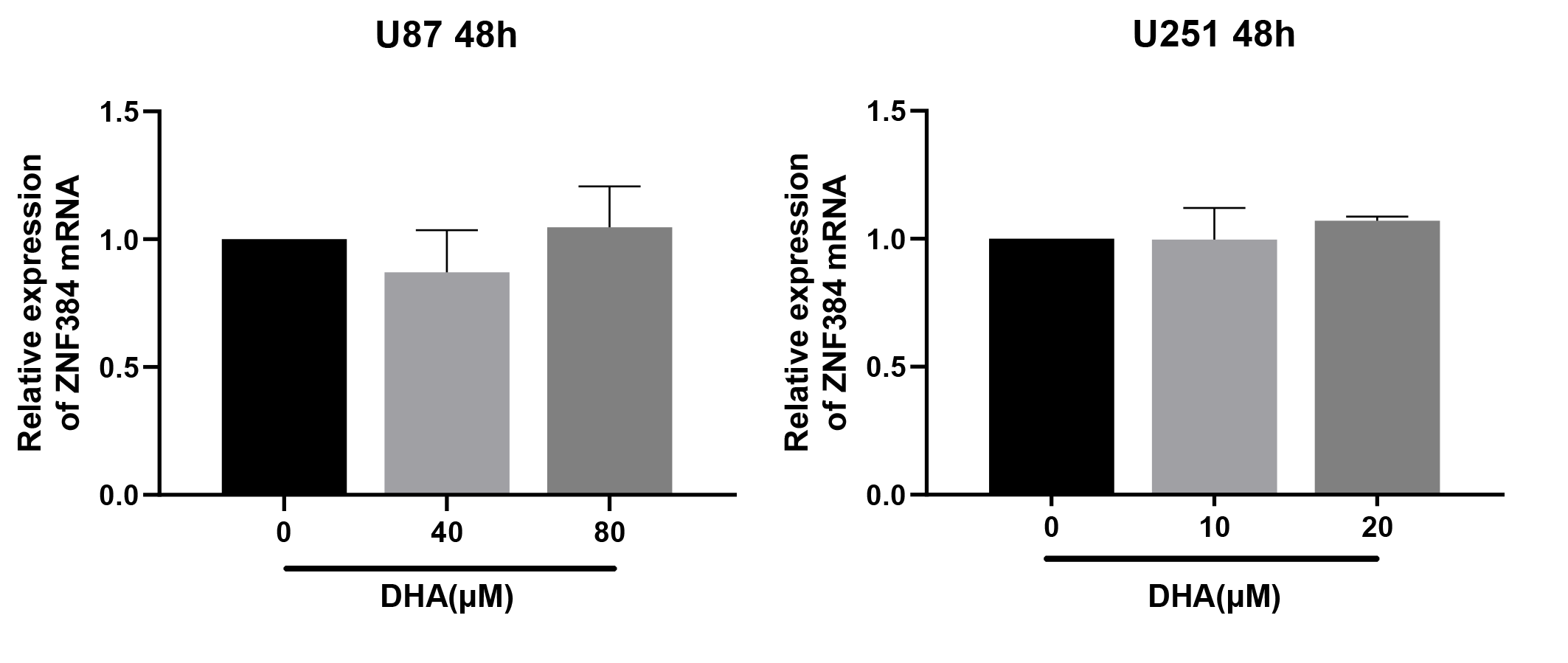
**

**Supplementary Figure 1.** The transcriptional expression level of ZNF384 in U87 and U251 cells were detected after DHA treatment with different concentrations for 48h by RT-qPCR.

**Supplementary Figure 2**

**
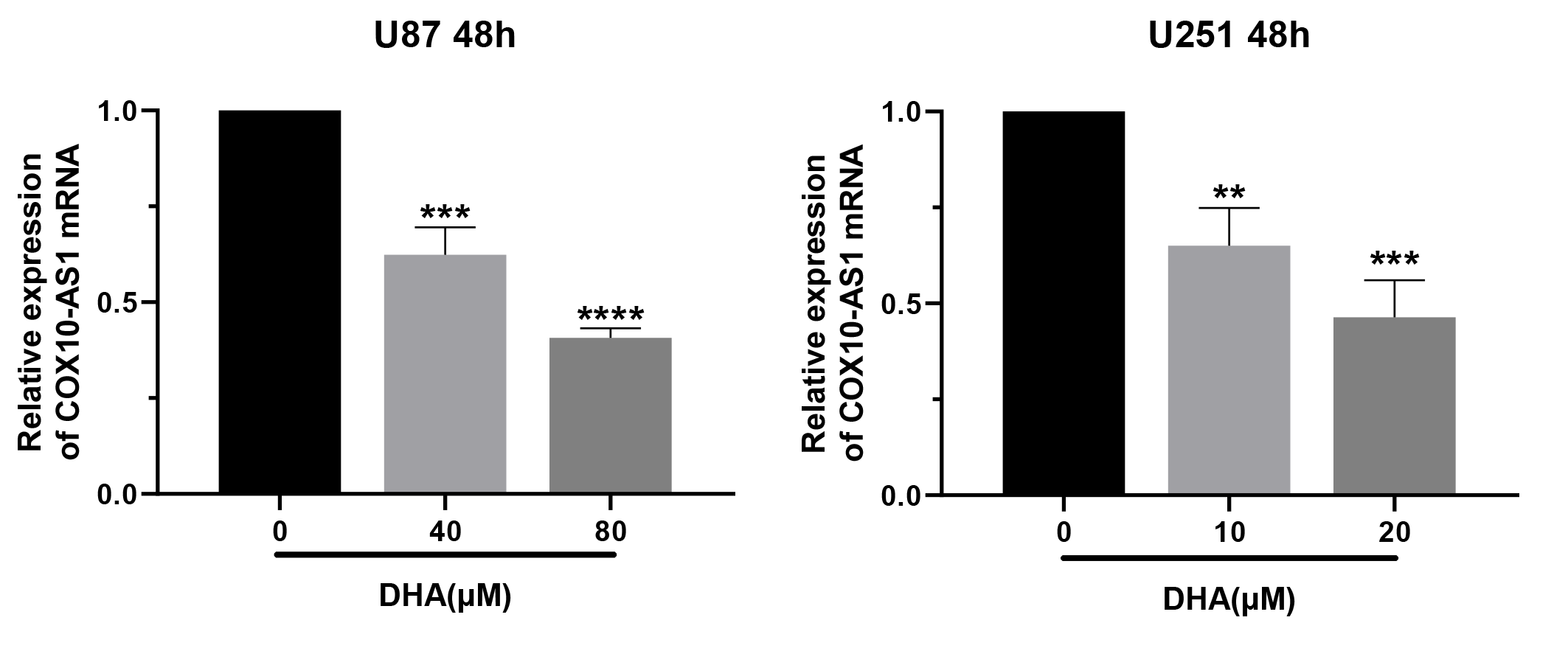
**

**Supplementary Figure 2.** The expression level of COX10-AS1 in U87 and U251 cells were detected after DHA treatment with different concentrations for 48h by RT-qPCR. **P<0.01, ***P<0.001,****P<0.0001; Data were mean ± SD from three independent experiments;n = 3 for all bar graphs.
